# 3D Printing of Hierarchically Porous Lattice Structures Based on Åkermanite Glass Microspheres and Reactive Silicone Binder

**DOI:** 10.3390/jfb13010008

**Published:** 2022-01-13

**Authors:** Arish Dasan, Jozef Kraxner, Luca Grigolato, Gianpaolo Savio, Hamada Elsayed, Dušan Galusek, Enrico Bernardo

**Affiliations:** 1 Centre for Functional and Surface-Functionalized Glass, Alexander Dubček University of Trenčín, 911 50 Trenčín, Slovakia; jozef.kraxner@tnuni.sk (J.K.); dusan.galusek@tnuni.sk (D.G.); 2 Department of Industrial Engineering, Università degli Studi di Padova, 35131 Padova, Italy; luca.grigolato@phd.unipd.it (L.G.); hamada.elsayed@unipd.it (H.E.); 3 Department of Civil, Environmental and Architectural Engineering (ICEA), University of Padova, 35131 Padova, Italy; gianpaolo.savio@unipd.it; 4 Refractories, Ceramics and Building Materials Department, National Research Centre, El Buhouth Str., Cairo 12622, Egypt; 5 Joint Glass Centre of the IIC SAS, TnUAD, and FChFT STU, FunGlass, Alexander Dubček University of Trenčín, 911 50 Trenčín, Slovakia

**Keywords:** bioceramics, åkermanite, glass microspheres, additive manufacturing, silicones

## Abstract

The present study illustrates the manufacturing method of hierarchically porous 3D scaffolds based on åkermanite as a promising bioceramic for stereolithography. The macroporosity was designed by implementing 3D models corresponding to different lattice structures (cubic, diamond, Kelvin, and Kagome). To obtain micro-scale porosity, flame synthesized glass microbeads with 10 wt% of silicone resins were utilized to fabricate green scaffolds, later converted into targeted bioceramic phase by firing at 1100 °C in air. No chemical reaction between the glass microspheres, crystallizing into åkermanite, and silica deriving from silicone oxidation was observed upon heat treatment. Silica acted as a binder between the adjacent microspheres, enhancing the creation of microporosity, as documented by XRD, and SEM coupled with EDX analysis. The formation of ‘spongy’ struts was confirmed by infiltration with Rhodamine B solution. The compressive strength of the sintered porous scaffolds was up to 0.7 MPa with the porosity of 68–84%.

## 1. Introduction

From a synthetic bone graft engineering perspective, the integration of multi-scale (macro-sized combined with micro- and nano-sized) porosity in 3D scaffolds is highly attractive [1,2]. Interconnected macropore networks (>100 μm) are essential for bone ingrowth, bone regeneration, and nutrient transport/waste evacuation, whereas micro- and nano-porosity aids cells adhesion and cell evolution [3]. At the same time, the mechanical properties of the grafted scaffolds are often compromised as the result of increased porosity. Notably, for load-bearing sites, a fine balance of mechanical support during degradation is important since the load should be slowly transferred to the regenerating bone tissue. Therefore, fine-tuning of these properties to achieve materials exhibiting a high strength-to-density ratio—with an optimal microstructure—is imperative, but still an open issue in the field of bone tissue engineering (BTE). 

Additive manufacturing (AM) technologies, in general, have a great potential for the obtainment of advanced bioceramic scaffolds featuring complex shapes. Ideally, the structure of macroporous scaffolds can be easily tailored using computer-aided design (CAD), subsequently transferred to several AM machines [4,5]. Recent efforts have been devoted to the development of micro/nanoporous structures in the same scaffolds through processing routes [6,7]. The simultaneous control of macro-porosity and micro/nano-porosity, however, remains an open issue.

The present investigation refers to åkermanite (Ca_2_MgSi_2_O_7_, i.e., 2CaO·MgO·2SiO_2_), one of the most interesting bioceramic systems studied in recent years. The use of bioceramics as bone scaffolds not only supports and stimulates new bone ingrowth but also provides mechanical support during tissue regeneration through mechano-transduction [8]. In the case of åkermanite, it has been found that the release of silicon, calcium, and magnesium ions as degradation by-products, with specific rates, play a vital role in the immune regulation and thereby fosters in situ tissue regeneration [9,10,11,12]. Again, an open issue consists of the coupling of advantages of åkermanite with the favourable topological features of hierarchically porous scaffolds mentioned above.

In our previous investigation, spherical glass particles with exact åkermanite stoichiometry were utilized to manufacture 3D scaffolds using the stereolithography AM technique [13]. Embedded into commercial acrylate precursors, microbeads allow photocurable slurries with much enhanced solid content, compared to irregular particles, with advantages in the integrity of scaffolds upon burn-out of the organic additive and densification upon viscous flow sintering. A remarkable struts densification, although favourable for the mechanical properties, is not desirable in the perspective of hierarchically porous scaffolds. Some limitations of viscous flow densification could come from the sudden increase of viscosity upon sintering, related to the crystallization of microbeads. This generally implies a need for careful control of heat treatment conditions. The present paper aims at the development of an alternative strategy, consisting of the exploration of silicone resin as a binder additive that yields upon heating a SiO_2_-rich phase. This phase ‘freezes’ the densification of glass microbeads that crystallize into the desired åkermanite phase.

## 2. Materials and Methods

Glass microbeads with åkermanite stoichiometric composition (Ca_2_MgSi_2_O_7_, i.e., 2CaO·MgO·2SiO_2_), manufactured by a flame synthesis technique, were used as the feedstock material. The detailed synthesis procedure was reported in our previous investigation [13]. High-quality liquid acrylate monomer (Prusa Resin-Tough, Original Prusa SL-1, Prusa Research a.s., Prague, Czech Republic) was used as UV photosensitive (405 nm) resin, supporting stereolithography 3D printing. A non-photocurable liquid silicone resin (H62C, Wacker-Chemie GmbH, Munich, Germany) was used as a preceramic polymer (ceramic yield = 58%) binder additive. Photocurable suspensions comprised microbeads (65 wt%) in photosensitive precursor with silicone resin added as 10 wt% of the total slurry weight.

**Figure 1:**
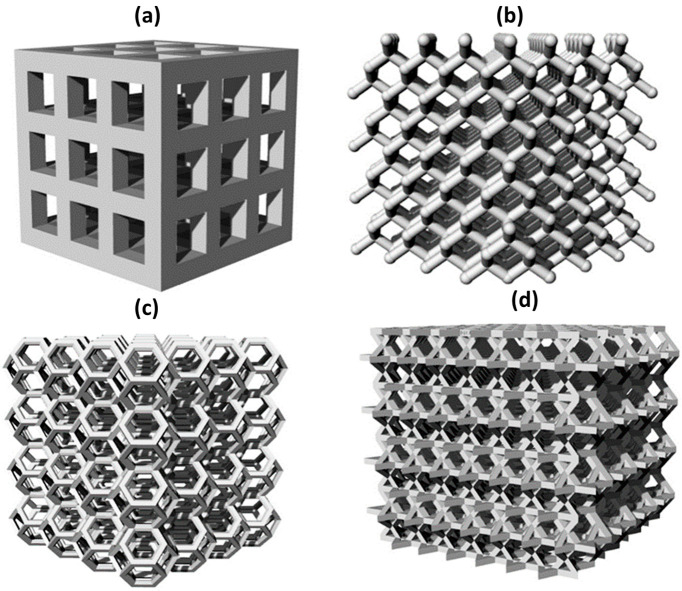
Lattice-based cellular assemblies adopted for MSLA scaffolds: (**a**) cubic; (**b**) diamond; (**c**) Kelvin; and (**d**) Kagome.

Four different lattice-based models (Cubic; number of cells in x, y and z direction: 3 × 3 × 3, cell dimension: 3 mm × 3 mm × 3 mm, strut thickness: 1 mm, theoretical relative density (TRD): 65%, Diamond; 4 mm × 4 mm × 4 mm, 7 mm × 7 mm × 7 mm, 0.8 mm, 85%, Kelvin; 4 mm × 4 mm × 4 mm, 12.7 mm × 12.7 mm × 12.7 mm, 1.6 mm, 92%, and Kagome; 4 mm × 4 mm × 4 mm, 8 mm × 8 mm × 8 mm, 1.2 mm, 72%) were designed, using the Rhinoceros 7 software, as shown in Figure 1, and used as a reference for the adopted masked stereolithography desktop printer (Original Prusa SL-1, Prusa Research s.r.o, Prague, Czech Republic), operating in the visible light range (between 400 and 500 nm). The adopted layer thickness was 50 µm, with an exposition lasting 7 s for each layer. The green scaffolds were subjected to debinding at 600 °C for 3 h, with a heating rate of 0.2 °C/min, followed by sintering at 1100 °C, both in the air and flowing nitrogen, for 1 h (heating rate 5 °C/min).

The mineralogical phases were identified using an X-ray powder diffractometer (Panalytical Empyrean, Malvern Panalytical, Eindhoven, The Netherlands) using Cu anode (Kα_1_ = 1.5406 Å and Kα_1_ = 1.5444 Å) equipped with a nickel Kβ filter. The diffraction data were evaluated using the software High Score Plus (v.3.0.4, Malvern Panalytical, Eindhoven, The Netherlands) supported by Crystallographic Open Database (COD_2013). 

Microstructural analysis on the sintered scaffolds was conducted by optical stereomicroscopy (AxioCamERc 5s Microscope Camera, Carl Zeiss Microscopy, Thornwood, New York, NY, USA) and scanning electron microscopy (SEM, JEOL 7600 F, JEOL Ltd., Tokyo, Japan) using an accelerating voltage of 20 kV. The semiquantitative chemical analysis was carried out using an Energy Dispersion X-Ray Spectrometer (EDS, Oxford Instruments, Oxford, UK) within Aztec systems (Oxford Instruments, Oxford, UK) with the ZAF (Z-atomic number, A-X-ray absorption, F-X-ray fluorescence) matrix correction method.

The geometrical density of the sinter-crystalized scaffolds was measured using a digital caliper and by weighting with an analytical balance. The apparent and true densities of the printed parts were measured by He pycnometry (Micromeritics AccuPyc 1330, Norcross, GA, USA), on bulk (3D printed scaffolds) and powdered samples, respectively. Total, open, and closed porosities were calculated according to the measured density values. Finally, the sintered scaffolds were impregnated with Rhodamine B solution (0.5% in 2-Propanol) to visualize the open porosity in the struts. The compressive strength of the fired scaffolds was measured using a universal testing machine (Quasar 25, Galdabini S.p.a., Cardano al Campo, Italy), operating with a cross-head speed of 0.5 mm/min.

## 3. Results and Discussion 

**Figure 2:**
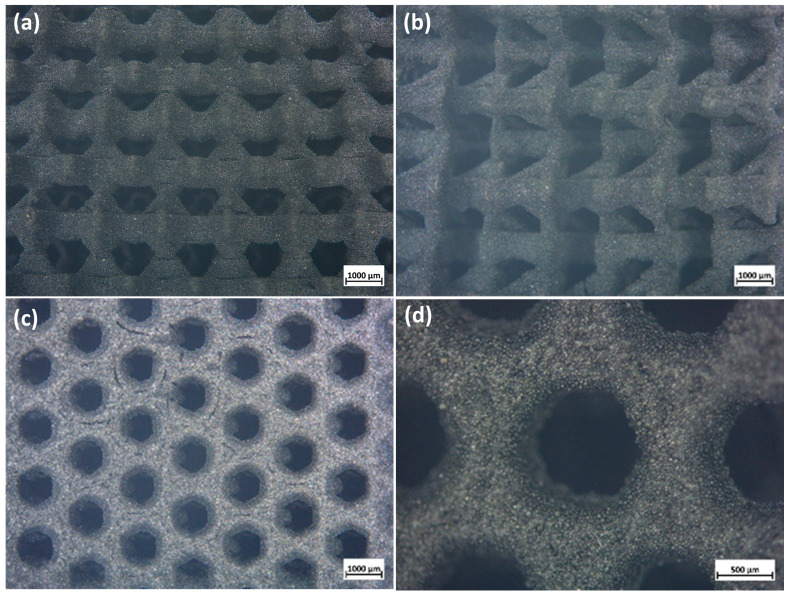
Optical images of selected 3D scaffolds (Kagome model); fired in nitrogen (**a**) front; (**b**) side; (**c**,**d**) top view.

Flame synthesis is particularly useful for the production of glass microspheres with controllable size (25–63 µm), especially in systems featuring a high crystallization rate such as åkermanite [13]. As mentioned previously, a primary aim was avoiding intensive viscous flow densification with shape loss, so that the hierarchical porosity could be determined simply by the packing of spheres. The binding of particles, after firing, had to be provided by the ceramic residue of the silicone additive. Silicones can be considered as reactive binders, since (i) they can be mixed with organic solvents and photocurable resin; (ii) they provide additional binding action, for un-sintered particles, at the burn-out of the organic compounds (at 500–600 °C), before high-temperature sintering; (iii) they contribute to the composition of the final ceramic [14,15]. Figure 2 confirms the feasibility of the fabrication of complex-shaped objects according to this approach.

**Figure 3:**
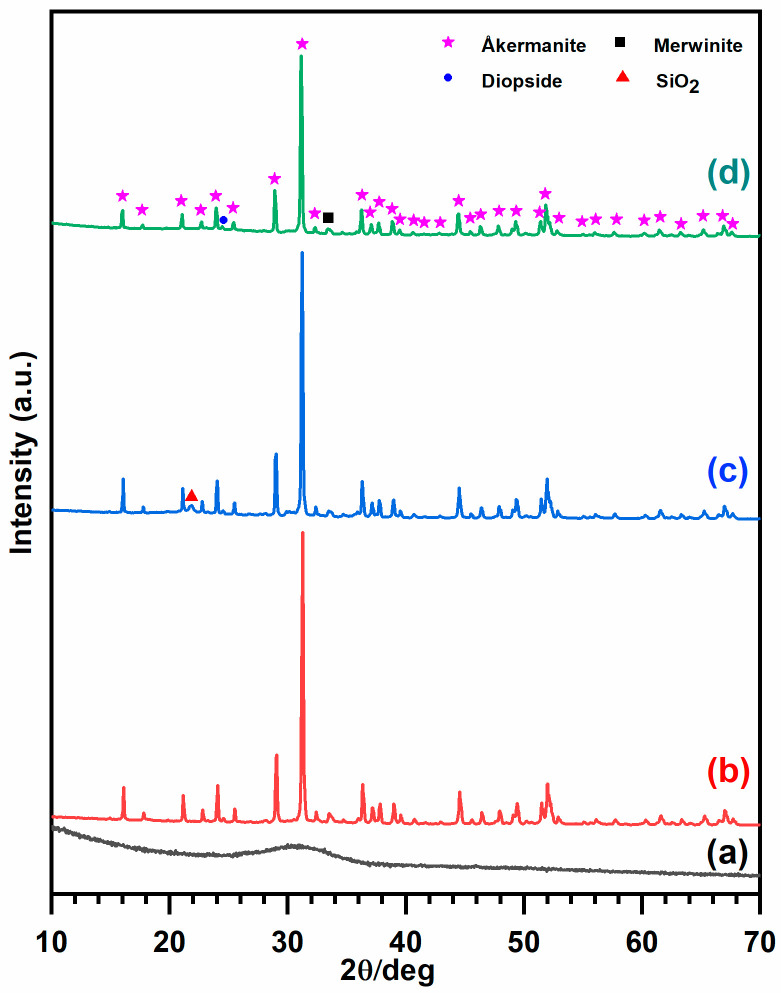
XRD patterns of (**a**) as-synthesized microspheres; (**b**) sintered scaffolds without silicone binder at 1100 °C in the air; (**c**) sintered scaffolds with silicone binder at 1100 °C in air; and (**d**) nitrogen.

The XRD pattern of as-synthesized microbeads, scaffolds fired at 1100 °C/1 h without (for comparison) and with silicone resins are presented in Figure 3. While the starting material was XRD amorphous (Figure 3a), the presence of åkermanite (PDF #83-1815), as the dominant crystalline phase (Figure 3b–d) in fired samples was confirmed. Additional diffraction maxima were attributed to traces of merwinite [Ca_3_MgSi_2_O_8_, PDF #89-2432] and diopside [CaMgSi_2_O_6_, PDF #75-1092] also known as excellent biomaterials [16]. 

**Figure 4:**
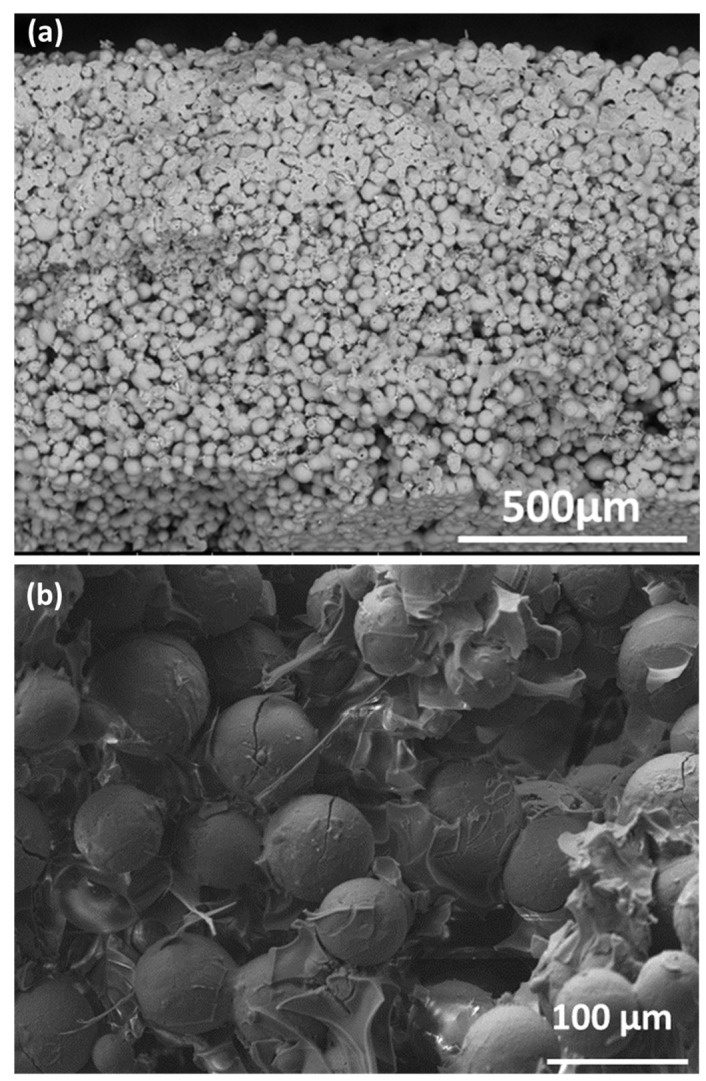
Low (**a**) and high (**b**) magnification SEM images of samples fired at 1100 °C in air.

**Figure 5:**
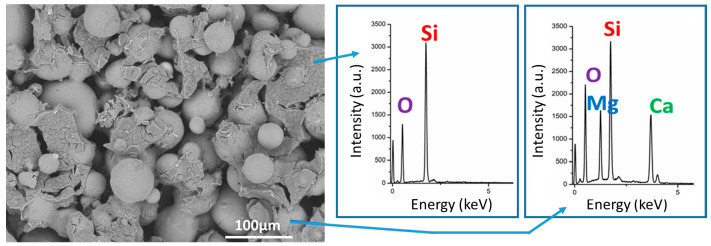
Higher magnification SEM micrograph and EDX spectra were recorded in the selected area of the scaffolds fired at 1100 °C in air.

Scaffolds fabricated using H62C silicone as binder additive, fired in the air, showed an additional peak centered at 2θ = 22° attributed to the presence of silicone-derived crystalline SiO_2_ [cristobalite, PDF #82-1014] (Figure 3c) [17]. Unlike in previous investigations on silicone/glass interaction [14,15], the resin and the glass microbeads did not chemically interact, and evolved independently. Additional evidence comes from the SEM images (Figure 4), showing that silica could act as a binder between adjacent microspheres. In particular, the high packing induced by using spherical particles is evident from Figure 4a. Silicone-derived material, as shown in Figure 4b, could at the same time bind the particles and prevent their coalescence, leaving a system of interconnected channels, visible also in Figure 5. The chemical composition of selected areas determined by energy-dispersive spectroscopy (EDS, Figure 5) reveals the distinction between microbeads (involving Ca and Mg) and silicone-derived binding phase (Ca- and Mg-free). 

**Figure 6:**
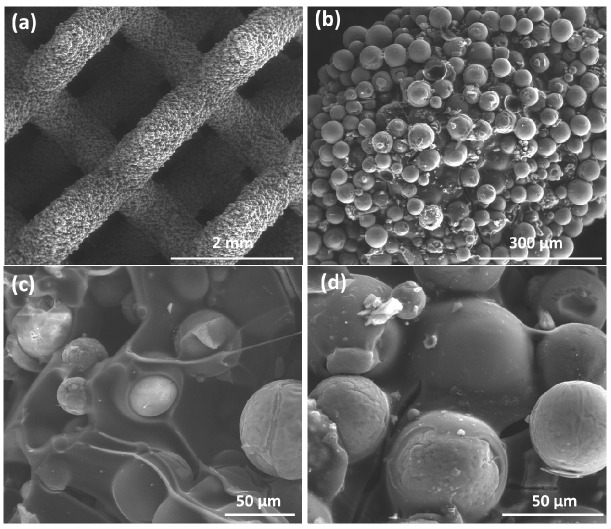
SEM images of samples fired at 1100 °C in nitrogen: (**a**) diamond cell structure; (**b**) packing of microbeads in a strut; (**c**–**d**) high magnification detail revealing the inclusion of microbeads in SiOC matrix.

In contrast, the scaffolds fired in flowing nitrogen (Figure 6a) did not show any cristobalite peak (Figure 3d). Again, crystallization only occurred within the microspheres while the silicone-derived binding phase remained amorphous, creating a silicon oxycarbide (SiOC) matrix. As documented by Figure 6b–d, the spherical particles were embedded in the SiOC matrix but not completely covered (due to the low content of SiOC). 

The fact that åkermanite crystallization was not affected by the silicone conversion into cristobalite or amorphous SiOC matrix is promising. The new systems may be seen as analogous to previously investigated CaCO_3_-silicone systems [18,19]. Unreacted, bioactive calcite could be embedded in inert silica or SiOC phase, having favorable biological response.

The poor sintering of glass microbeads, as a result of the presence of silica/SiOC intermediate layers, was expected to penalize the compressive strength, but specific topologies and firing conditions could provide certain compensation. Different cellular structures imply different stress distributions; compressive loading, in particular, corresponds to the bending or stretching of structural elements (depending on the specific model) [20].

**Figure 7:**
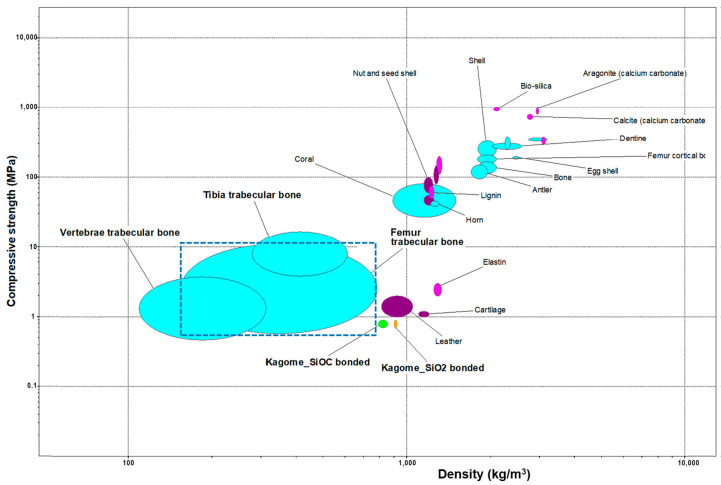
Ashby plot for biological materials and selected scaffolds [22].

The compressive strength measurements together with the porosity determinations are summarized in Table 1. The samples with the highest porosity showed a very low mechanical strength; some samples could be crushed by simple hand pressure, especially in the case of the Kelvin model, and are not considered in Table 1. However, changes in the design determined a remarkable increase of compressive strength, from 0.07 to 0.75 MPa. Such values are not directly comparable, since the total porosity, almost completely open, varied substantially (i.e., from 68 to 84%). A more reliable comparison comes from the calculation of the bending strength of the solid phase. According to the studies conducted by Gibson and Ashby [20], a highly porous ceramic lattice or sponge has a compressive strength ruled by:

σ_c_ ~ 0.2·σ_bend_·(ρ_rel_)^1.5^

where ρ_rel_ is the relative density (ρ_rel_ = 1 − P/100) and σ_bend_ is the bending strength of the solid phase positioned in the struts. Reversing the equation, σ_bend_* values could be obtained from experimentally determined values of compressive strength and porosity (see Table 1), which could be interpreted as the bending strength of the solid of a Gibson and Ashby lattice with the same compressive strength and porosity. The substantial differences (from 6 to 28 MPa) confirm the progress achieved through topological changes. The σ_bend_* values remain well below the bending strength of dense glass-ceramics (>100 MPa) [21], but the results from the Kagome structure are reputed as quite adequate for the application (at least in not-severely loaded implants). This can be easily understood from Figure 7, showing a compressive strength/density chart for biological materials (image and data from a commercial materials selection software package [22]). Any material is conventionally displayed as an ellipsis, with height and width defined according to minimum and maximum values of compressive strength and density; the statistical variations are actually better represented by a box. Especially SiOC-boned Kagome scaffolds approach m values of the femur trabecular bone.

The undoubtedly favorable effect of passing from an air to nitrogen atmosphere (increase of compressive strength from 21 to 28 MPa, for Kagome model) could be attributed to the suppression of the well-known transformation-induced cracking (upon cooling) of crystalline silica variants. The β→α-cristobalite phase transformation is the likely cause for the cracking of the silicone-derived binding phase in the samples fired in the air (as shown in Figure 5).

**Figure 8:**
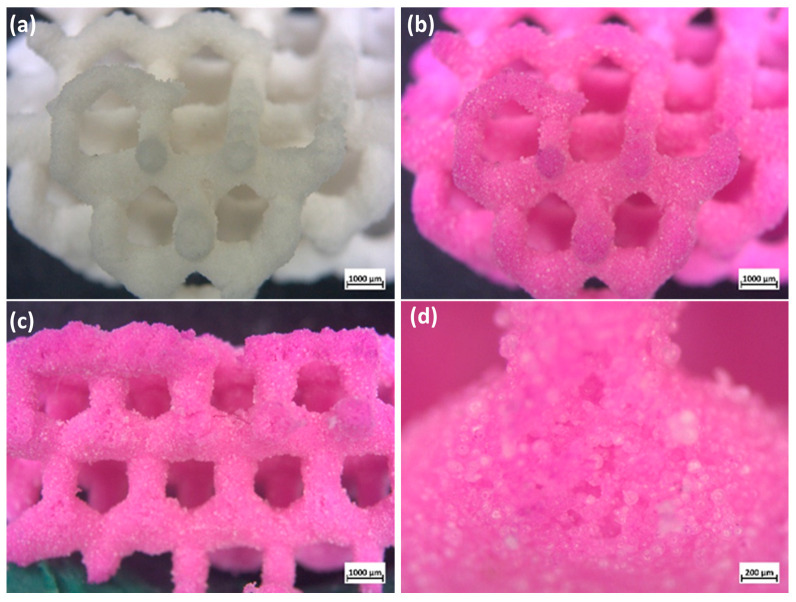
Optical images of (**a**) scaffolds (diamond lattice) fired at 1100 °C in air (before infiltration); (**b**) after infiltration in a Rhodamine B solution; (**c**,**d**) fracture surface (after infiltration).

The packing of microbeads, after partial sintering, effectively led to highly permeable materials, as verified by infiltration of rhodamine B solution. Figure 8 confirms that the dye molecules homogeneously dispersed and easily penetrated the entire scaffolds through the channels between adjacent particles. The coloration was thus observed not only at the surface but extended also to fracture surfaces. The obtained spongy scaffolds could therefore be potentially beneficial for enhancing biological performance in terms of cell attachment, growth, and differentiation, as well as vascularization. Infiltration of biopolymers could be also possible to form a new class of biocomposites with enhanced mechanical properties [23].

To further improve the mechanical strength, additional efforts will be undertaken in terms of topological control (e.g., by adopting triply periodic minimal surface structures (gyroids), or in terms of additional inclusion of silicone resin and modifications of firing conditions). Additional surface characterization techniques (such as contact angle determinations) are envisaged. Finally, in vitro and in vivo studies will also be performed to understand the biological performance of the sintered scaffolds.

## 4. Conclusions

The åkermanite based 3D scaffolds with multiscale porosity were manufactured by stereolitography technique. The main findings are summarized as follows:

- Spherical shaped glass particles were selected to fabricate the green scaffolds. The photocurable slurry comprises photosensitive resin, 65 wt% glass microbeads, and 10 wt% silicone resin, the latter found to impart structural integrity to the scaffolds, despite poor sintering of microbeads.
- The formation of åkermanite phase was dominant at the applied sintering temperature of 1100 °C. The firing conditions (firing in air or nitrogen) did not affect the crystallization of åkermanite, but affected the transformation of silicone resin into (partly crystallized) silica or fully amorphous SiOC.
- The change from silica to SiOC binding phase had a positive effect on the strength of the prepared scaffolds, by eliminating the formation of cristobalite. A more substantial effect on the compressive strength of scaffolds is associated with the adoption of different topologies.

## Figures and Tables

**Table 1:** Density and strength determinations.

3D Lattice Structures	Atm	Geometrical Density, ρ (g/cm^3^)	Total Porosity, P (vol %) [ρ_rel_ = 1 − P/100]	Open Porosity (vol %)	Compressive Strength, σ_c_ (MPa) [σ_bend_* (MPa)]
Diamond	air	0.46 ± 0.01	83.6 ± 0.3 [0.164]	83.5 ± 0.3	0.07 ± 0.01 [~6]
	N_2_	0.49 ± 0.01	84.1 ± 0.1 [0.159]	83.6 ± 0.1	0.08 ± 0.01 [~6]
Cubic	air	0.80 ± 0.09	72 ± 1 [0.229]	71.6 ± 0.1.4	0.5 ± 0.1 [~18]
	N_2_	0.75 ± 0.01	75.7 ± 0.8 [0.243]	75.3 ± 0.8	0.58 ± 0.05 [~24]
Kagome	air	0.91 ± 0.01	68 ± 2 [0.318]	67.9 ± 1.0	0.8 ± 0.1 [~21]
	N_2_	0.82 ± 0.03	73 ± 1 [0.269]	72.7 ± 1.0	0.8 ± 0.1 [~28]

## Data Availability

The data presented in this study are available on request from the corresponding author. The data are not publicly available due to privacy restrictions.
